# Bilateral reversed palmaris longus muscle: a case report and systematic literature review

**DOI:** 10.1007/s00276-019-02363-z

**Published:** 2019-11-12

**Authors:** Georga Longhurst, Danya Stone, Nick Mahony

**Affiliations:** 1 Anatomical Sciences, St George’s, University of London, London, UK; 2 Department of Anatomy, Brighton and Sussex Medical School, Brighton, UK; 3 Department of Anatomy, Trinity College, University of Dublin, Dublin, Ireland

**Keywords:** Palmaris longus, Reversed, Reverse, Inverted, Bilateral, Anatomical variation, Muscle

## Abstract

**Purpose:**

We present a case of a bilateral reversed palmaris longus muscle and a systematic review of the literature on this anatomical variation.

**Methods:**

Routine dissection of a 90-year-old male cadaver revealed a rare bilateral reversed palmaris longus. This was documented photographically, and length and relation to anatomical landmarks were recorded. This finding stimulated a systematic review of the literature on the reversed palmaris longus variation, from which measurements were collated and statistical analysis performed to determine the prevalence, average length, relationship to side and sex, and to discuss its clinical and evolutionary implications.

**Results:**

The average length of the muscle belly and tendon of reversed palmaris longus was 135 mm and 126 mm, respectively. Statistical analysis revealed no disparity in presentation due to sex and side; however, bilateral reversed palmaris longus has only been reported in males. A high proportion (70.8%) of reversed palmaris longus were discovered in the right upper limb compared to the left.

**Conclusion:**

Variations in palmaris longus are purported to be as a result of phylogenetic regression. Clinically, patients with this variant may present with pain or swelling of the distal forearm, often as a result of intense physical exertion related to occupation or sport. Clinicians should be aware of this muscle variant as its presence could lead to confusion during tendon allograft harvesting procedures in reconstructive and tendon grafting surgery.

## Introduction

Palmaris longus (PL) is a muscle of the anterior compartment of the forearm. It is located within the superficial group of flexor muscles, medial and superficial to pronator teres and flexor carpi radialis, and lateral to flexor carpi ulnaris. It is attached to the medial epicondyle and epicondylar ridge of the humerus via the common flexor tendon. Typically, the fusiform muscle belly runs distally and becomes tendinous in the mid-forearm. Tendinous fibers insert into the flexor retinaculum and palmar aponeurosis of the hand. Due to its insertion points, PL acts as an accessory flexor muscle and as an anchor of the skin and fascia to resist horizontal shearing forces. The neurovascular supply to PL is through the median nerve and branches of the ulnar artery [43].

Palmaris longus is considered to be the most variable muscle in human anatomy. The most common variation is complete agenesis, which is prevalent in 20.25% of the global population [49]. When present, PL can be digastric, duplicated, bifurcated, trifurcated or have atypical tendinous insertions [16]. While variations of PL are common, it is essential that rare presentations of the muscle are reported on, as these variations have important surgical and clinical implication. Firstly, PL is often used for tendon grafting and reconstructive surgeries as the removal of the muscle does not affect the function of the forearm and it is easily resected due to its superficial location [36]. The presence of a reversed PL (RPL) may prevent the tendon from being harvested for tendon grafting. Secondly, if present, RPL may be a rare cause of symptoms of neuropathy and neural compression, due to its close anatomical relationship to the median and ulnar nerves [15, 16].

## Materials and methods

### Case report

A 90-year-old male cadaver was fixed traditionally with 10% formalin. Skin and subcutaneous tissue of the forearms were reflected, and the antebrachial fascia and bicipital aponeuroses were removed. PL was observed to be in a reversed orientation bilaterally. RPL was separated from its fascial compartments and measurements of the tendons and muscle bellies were recorded with a 150 mm range digital-pocket-caliper (Scala, Germany). The proximal tendon was measured from the medial epicondyle of the humerus to the most proximal visible muscle fiber. The muscle bellies were measured from the most proximal to the most distal visible muscle fiber. The distal tendons were measured from the most distal muscle fiber to the tendon’s insertion point onto the palmar aponeurosis at a line between the proximal scaphoid and pisiform bones. Each measurement was duplicated and an average (mean) length was calculated.

### Literature review

A literature review was conducted using PubMed and Google Scholar databases, using the keywords: ‘reserved palmaris longus’ or ‘reverse palmaris longus’. Only papers which reported the presence of RPL were discussed. All related articles were carefully reviewed and studies that did not include information on side and sex were excluded from the study. The prevalence of RPL in surgical and cadaveric cases and the average length of the tendon and muscle belly was calculated. A Chi squared test with Fisher’s exact post hoc test was performed with GraphPad Prism (Version 6e) to assess the relationship of RPL to sex and side.

Primary consent was given by all donors in line with best practice guidelines developed by the Anatomical Committee of the Irish Medical Schools and the Medical Council of Ireland.

Research ethics committee approval for this study from the School of Medicine Research Ethics Committee at Trinity College Dublin was also obtained.

## Results

### Case report

**Fig. 1:**
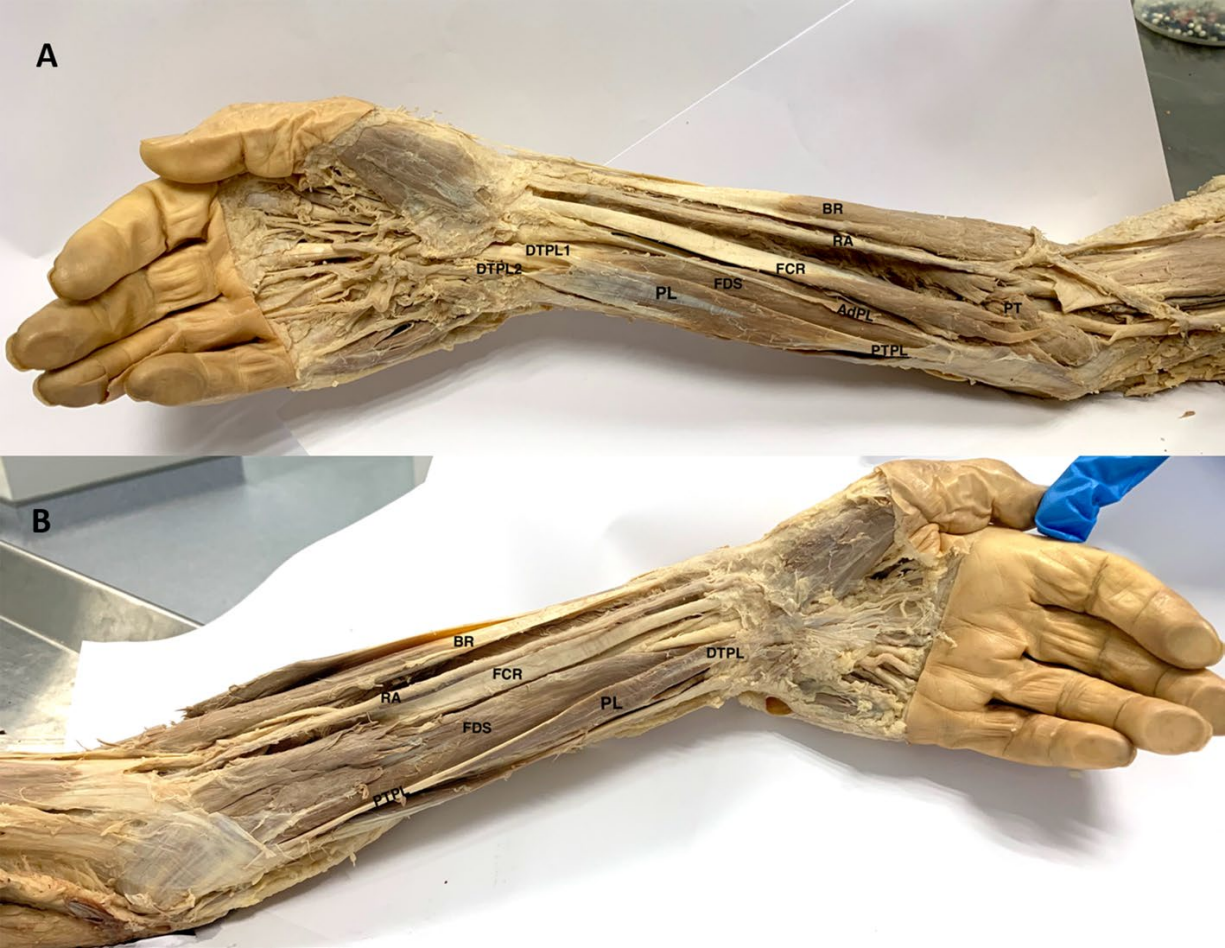
Dissection of right (**a**) and (**b**) left anterior compartment of the forearm demonstrating bilaterally reversed palmaris longus. *PL* palmaris longus, *PTPL* proximal tendon palmaris longus, *DTPL* distal tendon palmaris longus, *AdPL* additional slip of palmaris longus, *FDS* flexor digitorum superficialis, *FCR* flerox carpi radialis, *BR* brachioradialis, *PT* pronator teres, *RA* radial artery

Palmaris longus was reversed bilaterally with a proximal tendon, originating from the medial epicondyle of the humerus and distal muscle belly, inserting via a short distal tendon onto the flexor retinaculum and palmar aponeurosis of the hand. All other anatomical relations were consistent with the literature; however, the left RPL received an additional slip of muscle from flexor digitorum superficialis near its proximal insertion. Both arms showed no signs of muscular atrophy or hypertrophy. Proximally, the median nerve travelled deep to PL and there was no indication of median nerve entrapment along its course. Distally, the median nerve travelled more superficially in the carpal tunnel and entered the wrist lateral to RPL (Fig. 1).

The total lengths of the two (bilateral) RPL were 264 mm and 246 mm on the right and left forearms, respectively. The proximal and distal tendons were longer on the right side at 119 mm and 50 mm, respectively, compared to 116 mm and 11 mm on the left. Conversely, the muscle belly on the left was longer on the left side at 120 mm compared to 96 mm on the right (Table 1).

**Table 1:** Measurements of reversed palmaris longus muscle from case study

Right (mm)				Left (mm)			
DT	MB	PT	Total	DT	MB	PT	Total
50	96	119	264	11	120	116	246

*DT* distal tendon, *MB* muscle belly, *PT* proximal tendon

### Literature review

A total of 152 relevant articles were accessed from the years 1814 to 2019. Fifty-five cases of RPL were recorded in 39 of these articles (Table 2). Six articles were excluded after initial screening as they did not contain information on sex or side. In total, 33 articles were included that report. Thirteen (39.4%) variants were revealed through cadaveric studies, while 20 (60.4%) were reported in clinical case studies as a result of pain and swelling in the distal anterior forearm, and subsequent surgical intervention, or through ultrasound or magnetic resonance imaging (MRI).

**Table 2:** Prevalence of reversed palmaris longus in the literature

Study	No	Sex	Side	Loc	Clinical implications
Tenaw, 2018 [45]	1	M	R	CA	
Getzmann and Schweizer, 2018 [18]	1	F	R	CL	Competitive swimmer; swelling and pain of distal forearm with activity
George and Hassan, 2018 [15]	1	F	L	CL	Gymnast; swelling and pain of distal forearm
Bhashyam et al., 2017 [5]	1	M	L	CL	Laborer; exercise exacerbated, pain of distal forearm, ulnar nerve paresthesia
Twoon et al., 2017 [47]	2	F	L	CL	Competitive weightlifter; pain and swelling of distal forearm, exercise exacerbated symptoms
		M	R	CL	Keen sportsman; pain and swelling distal forearm, initially exercise exacerbated symptoms
Pires et al., 2018 [37]	1	M	R	CA	
Marpalli et al., 2016 [27]	1	M	R	CA	
Mathew et al., 2015 [28]	1	M	L	CA	
Eren et al., 2015 [9]	1	M	R	CL	Swelling of distal forearm
Gune et al., 2014 [21]	2	M	B	CA	
Heck and Campos, 2014 [23]	2	M	B	CA	
Murabit et al., 2013 [31]	1	F	L	CA	
Salgado et al., 2012 [39]	2	M	B	CA	
Cope et al., 2009 [7]	1	F	R	CA	Machine operator
Georgiev and Jelev, 2009 [17]	1	F	L	CA	
Acikel et al., 2007 [1]	1	M	R	CL	Soldier; swelling of distal forearm, exercise exacerbated pain, symptoms of median and ulnar nerve compression
Fazan., 2007 [12]	1	F	L	CA	Median nerve compression
Ogun et al., 2007 [34]	1	M	R	CL	Asymptomatic
Natsis et al., 2007 [32]	1	F	L	CA	
Seyhan, 2005 [42]	1	M	R	CL	Median nerve compression
Bencteux et al. 2001 [4]	1	F	R	CL	Pain and swelling of distal forearm
Yildiz et al. 2000 [50]	1	F	R	CL	Pain and swelling of distal forearm
Schuurman and van Gils, 2000 [41]	4	M	R	CL	All patients with effort-related median nerve compression
		F	R	CL	
		F	R	CL	
		F	R	CL	Swelling of distal forearm
Depuydt et al., 1998 [8]	2	F	R	CL	
		F	R	CL	
Ninkovic and Ohler, 1995 [33]	1	M	R	CL	Lumberjack; pain and swelling distal anterior forearm, median nerve paresthesia
Giunta et al., 1993 [19]	2	M	B	CL	Bilateral, median nerve compression, symptoms while working
Güler and Çeliköz, 1998 [20]	1	M	R	CL	Pain, numbness and tingling in thumb, index and middle finger
Regan et al., 1988 [38]	1	M	R	CL	Carpenter: Ulnar nerve compression with tingling of little finger. Blue swelling of wrist
Meyer and Pflaum, 1987 [29]	1	F	R	CL	Paresthesia in median nerve distribution, swelling of distal forearm
Schlafly and Lister, 1987 [40]	1	F	R	CL	Sales clerk; median nerve parenthesis with diffuse blue swelling. Weakness in thenar movement
Fragiadakis et al., 1978 [13]	3	M	R	CL	Median nerve compression
		M	R	CL	Asymptomatic
		F	R	CL	Asymptomatic
Still and Kleinert, 1973 [44]	3	M	R	CL	English professor; pain in volar aspect of right forearm that was accentuated by exercise, Median nerve paresthesia
		F	R	CL	Onset of pain when lifting heavy objects. Median nerve compression, thenar atrophy and decreased sensation in thumb, index and middle finger
		F	R	CL	Cashier; occasional numbness and tingling of entire hand.
Morrison, 1916 [30]	1	M	L	CA	Asymptomatic

*No* number, *M* male, *F* female, *R* right, *L* left, *B* bilateral, *Loc* location, *CA* cadaver, *CL* clinical

Taking our measurements, and the six reported in the literature into consideration, the average recorded length of the muscle belly and proximal tendon of RPL was 135 mm and 126 mm, respectively (Table 3). Of the 43 individuals where information on sex was available, 20 (46.5%) were female and 23 (53.5%) were male. Of the 48 upper limbs where information on side was available, 34 (70.9%) RPL presented in the right forearm, while 14 (29.1%) were found in the left. When isolating results from cadaveric studies, of 15 cases 7 (46.7%) were found in the right upper limb and 8 (53.3%) in the left. Five males (100%) presented with bilateral RPL. A Chi squared test with Fisher’s post hoc revealed no statistical difference in the frequency of presentation of RPL (*P* > 0.05) due to sex and side.

**Table 3:** Measurements of reversed palmaris longus from the literature

Study	Side	Sex	MB (mm)	PT (mm)
Gune et al., 2014 [21]	R	M	150	115
Gune et al., 2014 [21]	L	M	130	135
Heck and Campos, 2014 [23]	R	M	210	150
Heck and Campos, 2014 [23]	L	M	150	110
Georgiev and Jelev, 2009 [16]	L	F	136	89
Fazan, 2007 [12]	L	F	88	177
Case study	R	M	96	119
Case study	L	M	120	116
Mean			135	126
SD			37.9	27.1

*R* right, *L* left, *MB* muscle belly, *PT* proximal tendon

## Discussion

One of the earliest reports of RPL was discovered through dissection in 1868 at King’s College London [48]. The earliest clinical presentation, from our literature review, of bilateral RPL was reported in Germany in 1993 in a 21-year-old male who had suffered from median nerve compression, outside the carpal tunnel, as a result of hypertrophy of the muscle. Resection of the muscle belly relieved the symptoms immediately [19]. Three cases of bilateral RPL were reported in male donors from Chile [39], Brazil [23] and India [21]. All reports of RPL originated from the medial epicondyle of the humerus; however, the distal insertion points varied. One case reported on a short distal tendon on the left side, similar to our findings, which inserted into the distal aponeurosis [39]. Conversely, the remaining RPL attached to their distal insertion points via the distal muscular bellies directly [23]. Another case reported on a trifid insertion, which was laterally continuous with the fascia of the thenar muscles, centrally continuous with the palmar aponeurosis, and medially continuous with the fascia covering abductor digiti minimi [21]. The reported cases of RPL also showed differences in their relationship with the median nerve in the wrist. The right muscle and tendon were situated lateral to the median nerve, whereas the distal third of the left muscle compressed the median nerve [39].

There was no obvious correlation between sex and RPL. However, it is interesting to note that reports of bilateral cases of RPL have only been discovered in male donors to date, despite widespread anatomical dissection throughout the world. Perhaps this occurrence of RPL serves as an example of a minor evolutionary dimorphism between the sexes. A hypothesis warrants further investigations. Collating results from literature revealed a higher frequency of RPL in right upper limb. However, when only taking cadaveric studies into consideration, there is no difference in sidedness. This suggests that results for sidedness may not reflect the frequency of RPL in the population, but rather be skewed due to presentation in clinical scenarios. As RPL in right and left hand mirrors the population proportions of left and right handedness, this suggests that clinical presentations of symptoms of RPL result from increased activity of dominant limbs.

Previous studies have assessed the prevalence of bilateral agenesis of PL to sex and also reported no statistical correlation [26]. Conversely, a meta-analysis performed by Yammine et al. found that there is a statistically higher rate of agenesis on the right side compared to the left, but no significant difference between the combination of side and sex. In addition, the proportion of agenesis varies between different ethnic groups, with the lowest proportion among the East Asian and African population, and the highest observed in the Arab Middle Eastern population [49].

Only six sets of measurements of RPL muscle have been recorded in the literature from three previous studies [16, 20, 22]. As we report on a bilateral occurrence, we contribute two further measurements. Both tendon measurements were similar to those reported in the literature. The left tendon was 3 mm shorter than the average tendon measurement, whereas the right tendon did not deviate from the average (Table 2). Conversely, both muscle bellies were considerably shorter than the average measurement. The right muscle belly deviated 46 mm from the mean, while the left deviated 22 mm (Table 2).

Accurate measurements of PL are necessary as the muscle is often used in tendon grafting procedures to treat facial paralysis, ptosis correction and lip augmentation [36] and reconstructive surgeries [14]. According to Harvey and colleagues, a tendon must be greater than 150 mm for it to be suitable for tendon grafting [22]. We report that the average tendon length of RPL is 126 mm implying that this muscular anomaly is not suitable for tissue harvesting. Although it must be noted that a graft longer than 15 cm may not always be required depending on the type of reconstruction taking place [24]. Therefore, if possible ultrasound should be utilized to measure the tendon length and suitability for grafting pre-operatively. If ultrasound is not available, ineligibility of the muscle for use in tendon grafting may only be discovered intraoperatively [24, 25]. This may result in negative consequences for both the patient and surgeon as morbidity may increase and the patient may not have given consent for the harvesting of alternative tissue [11].

When discovered in a clinical scenario, RPL is often present in athletes or in professions that require intense physical exertion, such as lumberjacks, soldiers and manual laborers [14, 17, 48]. The repeated action of axial loading, power gripping and flexion/extension stresses can cause the muscle belly of the RPL to hypertrophy. The additional volume and pressure in the flexor compartment of the forearm may cause exertional compartment syndrome resulting in median or ulnar nerve paranesthesia and effort-related volar forearm pain [1, 8, 39]. Clinicians, physiotherapists and radiologists should be aware of this rare muscular anomaly, as it may be overlooked when diagnosing forearm pain and could be mistaken for a tumor or ganglion on MRI or clinical examination [4, 17].

Anatomical variations in the PL muscle are a consequence of phylogenetic degeneration [43]. This occurred as a result of a shift from quadrupedal or arboreal locomotion to habitual bipedalism during human evolution. As the function of the forearm evolved from a weight-bearing forelimb to a prehensile limb, capable of precise movement, the flexor muscles have partially atrophied in a caudocranial direction [2]. In fact, the relative tendon size of PL increases from ancestral genera, such as lemuriformes and new world primates, to more derived genera such as great apes including modern humans [3]. This shortening of the muscular belly has resulted in an overall weakening of the muscle and, as a result, complete agenesis of the PL does not affect forearm function. The anatomical variations of PL and its rudimentary function have led to the hypothesis that the upper extremity in man is still undergoing a process of evolution [6, 10].

Variations of the PL muscle are caused by disturbances of the intracellular or extracellular pathways that control embryonic muscle growth, which cannot be compensated for during later development. Anomalies and agenesis of PL in human fetuses are similar to those observed in adults [35]. Interestingly, variations in PL are distinctly heritable. Thomson et al. hypothesized that partial or complete absence of the muscle is due to a single dominant gene, which acts as an inhibitor of its development, but this inhibition is frequently incomplete due to modifying conditions such as sex and side [46].

Although a search strategy was implemented, it cannot be assumed that all articles containing information on this variant were included. However, efforts were made to include the most relevant studies containing the most information relevant to this review.

## Conclusion

Variations in the PL muscle are not uncommon and are purported to be as a result of phylogenetic regression. We have presented a further rare bilateral anomaly of a RPL again in male cadaver. Clinicians should be aware of this muscle variant as it may be a rare cause of volar wrist pain in grip-intensive sports or occupations and may give rise to confusion in reconstructive and plastic surgery.
